# Identification and Characterization of Novel Small RNAs in *Rickettsia prowazekii*

**DOI:** 10.3389/fmicb.2016.00859

**Published:** 2016-06-08

**Authors:** Casey L. C. Schroeder, Hema P. Narra, Abha Sahni, Mark Rojas, Kamil Khanipov, Jignesh Patel, Riya Shah, Yuriy Fofanov, Sanjeev K. Sahni

**Affiliations:** ^1^Department of Pathology, University of Texas Medical BranchGalveston, TX, USA; ^2^Department of Pharmacology, University of Texas Medical BranchGalveston, TX, USA; ^3^Department of Neuroscience, University of Texas at DallasDallas, TX, USA

**Keywords:** *Rickettsia prowazekii*, small RNAs, RNA sequencing, vascular endothelium, epidemic typhus

## Abstract

Emerging evidence implicates a critically important role for bacterial small RNAs (sRNAs) as post-transcriptional regulators of physiology, metabolism, stress/adaptive responses, and virulence, but the roles of sRNAs in pathogenic *Rickettsia* species remain poorly understood. Here, we report on the identification of both novel and well-known bacterial sRNAs in *Rickettsia prowazekii*, known to cause epidemic typhus in humans. RNA sequencing of human microvascular endothelial cells (HMECs), the preferred targets during human rickettsioses, infected with *R. prowazekii* revealed the presence of 35 trans-acting and 23 cis-acting sRNAs, respectively. Of these, expression of two trans-acting (*Rp*_sR17 and *Rp*_sR60) and one cis-acting (*Rp*_sR47) novel sRNAs and four well-characterized bacterial sRNAs (RNaseP_bact_a, α-tmRNA, 4.5S RNA, 6S RNA) was further confirmed by Northern blot or RT-PCR analyses. The transcriptional start sites of five novel rickettsial sRNAs and 6S RNA were next determined using 5′ RLM-RACE yielding evidence for their independent biogenesis in *R. prowazekii*. Finally, computational approaches were employed to determine the secondary structures and potential mRNA targets of novel sRNAs. Together, these results establish the presence and expression of sRNAs in *R. prowazekii* during host cell infection and suggest potential functional roles for these important post-transcriptional regulators in rickettsial biology and pathogenesis.

## Introduction

As critical post-transcriptional regulators of gene expression, regulatory RNAs have now been found in a wide array of organisms from all branches of life and considered to be ubiquitous in nature. Small regulatory RNAs (sRNAs) in pathogenic bacteria have garnered immense recent attention due to their ability to control diverse, physiologically important lifecycle processes such as quorum sensing, metabolism, stress responses, and virulence. Typically ranging from 50 to 500 nucleotides in length, sRNAs are heterogeneous in size and structure. Despite being longer in length, these sRNAs are considered to be analogous to eukaryotic sRNAs in the context of certain functional implications. Post-transcriptional sRNA-mediated regulatory mechanisms are broadly categorized into sRNA-protein and sRNA-mRNA interactions. Interactions with the protein-coding transcripts are further categorized into two groups, namely, trans-acting, and cis-acting (Liu and Camilli, 2010; Gottesman and Storz, 2011). Trans-acting sRNAs are defined as those encoded within the intergenic regions of a bacterial genome and act on target RNAs located elsewhere in the genome. On the other hand, cis-acting sRNAs originate from the antisense strand of an open reading frame (ORF) and tend to exert direct regulatory influence on that particular ORF. A hallmark feature of cis-acting sRNAs, therefore, is nearly perfect nucleotide complementarity to the target genes, unlike only partial nucleotide complementarity displayed by trans-acting sRNAs. Due in part to this partial complementarity, trans-acting sRNAs generally require the involvement of an RNA chaperone to facilitate nucleotide binding (Waters and Storz, 2009).

Based on a combination of detailed molecular phylogenetics, antigenic and proteomic profiles, epidemiologic and ecologic investigations of arthropods as transmitting vectors, and disease presentation, obligate intracellular bacteria in Genus *Rickettsia* are now divided in four groups—ancestral, spotted fever, transitional, and typhus. Epidemic typhus due to *R. prowazekii* is transmitted by the body lice (*Pediculus humanus corpis)* and has historically been dubbed as the scourge of armies due to massive outbreaks in the times of wars until World War I. Because of the precedent and possibility of its use as a potential bioweapon, *R. prowazekii* is also classified as a select agent. In humans, endothelial cells lining the small and medium-sized blood vessels are the primary targets of infection and illness is characterized by progressive endothelial damage leading to widespread vascular dysfunction and enhanced permeability from the intravascular compartment to the interstitium and culminating in generalized vasculitis (Bechah et al., 2008; Walker and Ismail, 2008). Without proper antibiotic treatment, the mortality rate for epidemic typhus, caused by *R. prowazekii*, reportedly ranges from 10 to 60% making it one of the most severe human rickettsioses (Raoult et al., 2004; Bechah et al., 2008). In addition, the disease can reappear in completely recovered patients years after the initial infection as a distinct clinical syndrome called recrudescent typhus or Brill-Zinsser disease (Parola and Raoult, 2006).

Sequencing of a pathogen's genome is an effective umbrella approach to identify unknown genotypic/phenotypic traits and to establish a platform for dissecting and deciphering gene function. Ready availability of a number of rickettsial genomes has had a dramatic impact on our understanding of their genetic diversity, genomic architecture, gene identification and function, and mechanisms of pathogenesis. As the first rickettsial genome to be sequenced and published (Andersson et al., 1998), *R. prowazekii* was found to carry a rather high amount (~24%) of non-coding DNA (Andersson et al., 1998; Holste et al., 2000) and an AT rich genome with a GC content of 29.1%, suggesting genomic reduction and gene neutralization due to obligate intracellular parasitism. While intergenic regions in other bacteria harbor small non-coding RNAs, their presence in *Rickettsia* species remained an open question until recently, when we predicted a number of sRNAs in rickettsial genomes using complementary computational approaches (Schroeder et al., 2015). Using infection of human microvascular endothelial cells (HMECs) with *R. prowazekii* as an experimental model system, we further determined the presence of six novel trans-acting sRNAs, but a limitation of this study was the exclusion of potential cis-acting sRNAs and possibly other novel trans-acting sRNAs. Here, we report on the identification, validation, and characterization of both cis-acting and additional novel trans-acting sRNAs in *R. prowazekii*. We have identified 35 novel trans-acting and 23 cis-acting sRNAs through next generation sequencing and confirmed the expression of four novel sRNAs in addition to well-known noncoding sRNAs, namely, α-tmRNA, RNaseP_bact_a, *ffs*, and 6S RNA. We further analyzed our validated sRNAs using experimental and bioinformatics techniques to determine their transcriptional start sites, upstream promoter motifs, and potential target genes.

## Materials and methods

### *Rickettsia* and cell culture

Human dermal microvascular endothelial cells (HMECs) were cultured in MCDB131 medium supplemented with L-glutamine (10 mmol L^−1^), epidermal growth factor (10 ng mL^−1^), hydrocortisone (1 μg mL^−1^), and 10% heat-inactivated fetal bovine serum and grown at 37°C with 5% CO_2_ until ~80 to 90% confluency (Rydkina et al., 2010). The use of human cell lines in our study was exempt by the University of Texas Medical Branch (UTMB) Institutional Review Board (IRB), but approved by the UTMB Institutional Biosafety Committee (IBC). Stocks of *R. prowazekii* strain Breinl were prepared by infecting Vero cells in culture, followed by purification of rickettsiae by differential centrifugation. The titers of infectious stocks were estimated by using a combination of quantitative PCR using primer pair *Rp*877p-*Rp*1258n for citrate synthase gene (*gltA*) and plaque assay (Roux et al., 1997; Rydkina et al., 2010). HMECs were infected with ~6 × 10^4^ pfu of rickettsiae per cm^2^ of culture surface area under BSL-3 conditions to achieve an MOI of 5:1 (an average of five or six intracellular rickettsiae per cell and infection of a majority [>80%] of cells (Rydkina et al., 2005, 2007, 2010). To allow for efficient rickettsial adhesion and invasion, the cells were incubated for 15 min with gentle rocking with initial infectious inoculum containing *R. prowazekii* in culture medium prior to replacement with fresh medium. Infected cells were then incubated at 37°C with 5% CO_2_ until processing for the isolation of DNA and RNA.

### RNA isolation and sequencing

For deep sequencing, HMECs infected with *R. prowazekii* for 3 and 24 h were subjected to the isolation of total RNA using our standard Tri-Reagent (Molecular Research Center) protocol. The RNA samples were treated with DNaseI (Zymo Research) to ensure removal of any contaminating genomic DNA and further processed sequentially through MICROBEnrich (Ambion) and Ribo-Zero (Epicentre) kits to remove interfering eukaryotic mRNAs and ribosomal RNAs, respectively. The enriched RNA preparations thus obtained were quantified using the MultiSkan Go Microplate Spectrophotometer (ThermoScientific) and assessed for the quality control on an Agilent 2100 Bioanalyzer (Agilent Technologies). Two independent cDNA libraries from enriched but non-size selected RNA samples for each experimental condition were then constructed using the TruSeq RNA Sample Prep Kit (Illumina) as per manufacturer's directions. Strand-specific sequencing was carried out on an Illumina HiSeq 1500 instrument at our institutional Next Generation Sequencing Core facility. The sequencing libraries were comprised of 50 bp long reads in a FASTQ format. The quality of each read was assessed and any base with a PHRED score of 15 or below was excluded from the analysis. In addition, the first 14 bases of each read were trimmed and any reads mapping to the human genome (version GRCh38/hg38) were excluded. The remaining 36 bp long reads were then mapped onto the *R. prowazekii* Breinl genome (NC_020993) allowing up to two base mismatches using Bowtie2 (Langmead and Salzberg, 2012). For each candidate novel sRNA, the average read coverage for each nucleotide was normalized to the length of the predicted sRNA. The same was also computed for 50 nucleotides up- and down-stream of each prediction. The Mean Expression Value (MEV) was then calculated by computing the ratio between the predicted sRNA and the flanking nucleotides (Raghavan et al., 2011; Warrier et al., 2014). An MEV cutoff value of ≥5x was used throughout this work.

### Northern blotting

HMECs infected with *R. prowazekii* as described above were processed for total RNA isolation using Tri-Reagent (Molecular Research Center) according to our standard protocol. The RNA samples were then treated with DNaseI (Zymo Research) and subjected to the MICROBEnrich Kit (Ambion). The RNA thus obtained were further purified via precipitation with 100% v/v ethanol prior to the samples determination of concentration in final preparations using a MultiSkan GO Microplate Spectrophotometer (ThermoScientific).

Northern blot analysis was carried out using the NorthernMax kit (Ambion) following the manufacturer's instructions. Enriched bacterial RNA (15 μg per lane) was loaded onto a 1.5% agarose-formaldehyde gel, electrophoresed at 90 V, and then transferred onto a Zeta-Probe Blotting Membrane (Bio-Rad). Membranes were cross-linked using UV Stratalinker 1800 (Stratagene). A PCR template with a T7 promoter on the anti-sense strand for the sRNA under investigation was created using GoTaq® Green DNA Polymerase (Promega). Using the PCR template, strand-specific, [α-^32^P] UTP-labeled RNA probes were synthesized through *in vitro* transcription with the MAXIscript® kit (Ambion) (Supplemental Table S2). Each RNA probe was treated with DNase I for 15 min at 37°C as per manufacturer's directions to remove the original PCR template. Unincorporated nucleotides were removed using Illustra MicroSpin G-25 Columns (GE Healthcare). Membranes were hybridized overnight at 50 to 65°C depending on the probe sequence, washed thoroughly using standard Northern wash solutions, and then exposed to autoradiography film.

### Reverse transcriptase PCR

One microgram (1 μg) of DNase I treated total RNA was reverse transcribed using SuperScript® VILO cDNA Synthesis Kit (Life Technologies) with random hexamers following manufacturer's instructions. Reverse transcriptase PCR was performed using GoTaq® Green Master Mix Kit (Promega). Each 25 μL reaction contained a final concentration of 1X GoTaq® Master Mix (contains DNA polymerase and dNTPs), 0.5 μM forward primer, 0.5 μM reverse primer, and 100 ng cDNA template. Thermal cycler conditions were: stage 1 at 95°C for 5 min, stage 2 (35 cycles) at 95°C for 30 s, 60°C for 30 s, and 72°C for 30 s, and stage 3 at 72°C for 10 min. Samples were separated on a 2% agarose gel, stained with ethidium bromide, and imaged on ChemiDoc MP imaging system (Bio-Rad). Primers are listed in Supplemental Table S2.

### RNA ligase-mediated rapid amplification of cDNA ends (RLM-RACE)

The 5′ sRNA sequence was determined using FirstChoice® RLM-RACE kit (Ambion) according to the manufacturer's instruction manual. Ten micrograms of DNase-treated, enriched RNA was incubated with tobacco alkaline phosphatase (TAP) for 1 h at 37°C. The 5′ adaptor sequence was then ligated to TAP-treated RNA at 37°C for 1 h. Reverse transcription reaction was carried out using random decamers at 42°C for 1 h. Nested PCR was conducted with necessary modifications to the manufacturer's directions. In order to optimize the cycling conditions yielding consistent amplification, gradient PCR reactions were performed using both the outer and inner primer pairs. Thus, optimal conditions yielding the cleanest and strongest product for each sRNA were employed in all assays. Primers are listed in Supplemental Table S3. PCR products were cloned into the pGEM-T Easy vector (Promega). Sanger Sequencing was conducted at the institutional Molecular Genomics Core.

### Promoter prediction

Using the web-based software BPROM, bacterial promoters were predicted for each of the sequenced rickettsial noncoding RNAs by searching for −10 box and −35 box corresponding to the σ-factor promoter, transcription factor binding sites, and a transcription start site (Solovyey and Salamov, 2010). Overall, BPROM has been reported to have 80% accuracy and specificity (Solovyey and Salamov, 2010). Each promoter prediction was conducted using 150 base pairs upstream of the predicted sRNA. Nucleotide frequency plots were created using the −10 box and −35 box predictions. The web based program WebLogo3 from the University of California at Berkeley was used to generate the sequence logos (Crooks et al., 2004).

### Target prediction

Target genes for each candidate sRNA were predicted using two independent web based programs, TargetRNA2 and IntaRNA (Busch et al., 2008; Tjaden, 2008). TargetRNA2 searches a genome's annotated features for a statistically significant basepair-binding potential to the queried nucleotide input. The individual basepair model was used throughout target predictions. The program calculates a hybridization score followed by a statistical significance of each potential RNA interaction (Tjaden, 2008). The following parameters were used for each prediction. For statistical significance, the *P*-value was set at ≤0.05. The program searched 80 nucleotides before the start codon and 20 nucleotides after the start codon. The seed length was 7 consecutive nucleotides and corresponds to the average seed length (6 to 8 nucleotides) for trans-acting sRNAs (Gottesman and Storz, 2011). The filter size, which corresponds to how the program filters out non-target mRNAs, was set at the default value of 400. Conversely, IntaRNA assesses the query sRNA nucleotide sequence with the selected genome and calculates a combined energy score from the free energy hybridization and interaction sites. This program has less customizable features than TargetRNA2. For these predictions, the parameters were set to default with a minimum number of 7 base pairs in the seed region. For statistical significance, the *P*-value was set at ≤0.05. Any targets with a *P* > 0.05 were discarded from the analysis.

## Results

### Identification of *R. prowazekii* sRNAs through RNA sequencing

In a recently published study, we have predicted the existence of 26 candidate sRNAs within the *R. prowazekii* strain Breinl genome using a web-based interface SIPHT. Upon further analysis, 12 of these candidates were found to have an MEV of >1.5 and six were confirmed through RT-PCR (Table 1) (Schroeder et al., 2015). A limitation of this study, however, was that the programs such as SIPHT only survey the intergenic regions and do not screen the regions antisense to ORFs, thus failing to identify cis-acting sRNAs. To investigate the presence of cis-acting sRNAs and to perform a deeper analysis of the transcriptome to identify additional trans-acting sRNAs that do not meet the parameters of the SIPHT program, we have now performed next generation sequencing to identify and catalog all sRNAs in *R. prowazekii*.

**Table 1:** **List of small RNAs found within the *Rickettsia prowazekii* genome**.

***Rp_sR*^a^**	**Approximate start**	**Approximate stop**	**Size (bp)**	**Strand**	**Type of sRNA**	**Homology**	**Strand orientation**	**Notes**	**References**
1	10482	10278	204	R	Trans	TG	</</<	Predicted as SIPHT #22	Schroeder et al., 2015
2	10774	11003	230	F	Trans	Rp	</>/<	Identified by RNA-seq	This study
3	14215	13984	232	R	Cis	Rp	H375_160	Identified by RNA-seq	This study
4	15459	15203	257	R	Cis	TG, TRG, SFG	H375_160	Identified by RNA-seq	This study
5	19850	19622	229	R	Trans	TG, TRG	>/ < />	Identified by RNA-seq	This study
6	22107	22298	192	F	Trans	Rp	</>/>	Identified by RNA-seq	This study
7	24935	25206	272	F	Cis	TG, TRG, SFG	H375_230	Identified by RNA-seq	This study
8	47692	47542	150	F	Trans	Rp	>/>/>	Predicted as SIPHT #21	Schroeder et al., 2015
9	48035	48200	166	F	Trans	Rp	>/>/>	Identified by RNA-seq	This study
10	48620	48834	215	F	Trans	Rp	>/>/>	Identified by RNA-seq	This study
11	71739	72043	305	F	Cis	TG, TRG, SFG	H375_570	Identified by RNA-seq	This study
12	76189	76452	264	F	Cis	AG, TG, TRG	H375_570	Identified by RNA-seq	This study
13	77115	77335	221	F	Trans	Rp	</>/<	Identified by RNA-seq	This study
14	78259	78513	255	F	Trans	Rp	</>/>	Identified by RNA-seq	This study
15	88423	88616	194	F	Trans	Rp	>/>/ <	Identified by RNA-seq	This study
16	100282	99698	585	R	Cis	TG, TRG, SFG	H375_740	Identified by RNA-seq	This study
17	105748	105486	263	R	Trans	Rp	>/ < />	Identified by RNA-seq	This study
18	116363	116200	164	R	Cis	TG	H375_870	Identified by RNA-seq	This study
19	132593	132781	189	F	Trans	Rp	</>/>	Identified by RNA-seq	This study
20	163641	163556	85	R	Trans	AG, TG	</</>	Predicted as SIPHT #2	Schroeder et al., 2015
21	173793	173974	182	F	Cis	TG, TRG, SFG	H375_1300	Identified by RNA-seq	This study
22	177789	178118	330	F	Cis	TG, SFG	H375_1330	Identified by RNA-seq	This study
23	185267	185484	218	F	Cis	TG, TRG, SFG	H375_1400	Identified by RNA-seq	This study
24	218432	218657	226	F	Cis	TG, TRG, SFG	H375_1610	Identified by RNA-seq	This study
25	257587	257856	270	F	Cis	TG, TRG, SFG	H375_1930	Identified by RNA-seq	This study
26	261134	261341	208	F	Trans	Rp	>/>/ <	Identified by RNA-seq	This study
27	262322	262601	280	F	Trans	Rp	>/>/ <	Identified by RNA-seq	This study
28	275336	275043	294	R	Cis	TG, TRG	H375_2050	Identified by RNA-seq	This study
29	306070	305924	146	F	Trans	Rp	>/>/>	Predicted as SIPHT #12	Schroeder et al., 2015
30	308324	308042	282	R	Trans	TG	>/ < / <	Predicted as SIPHT #11	Schroeder et al., 2015
31	308652	308848	197	F	Trans	Rp	>/>/ <	Identified by RNA-seq	This study
32	309308	309564	257	F	Cis	TG, TRG, SFG	H375_2380	Identified by RNA-seq	This study
33	315408	315772	365	F	Cis	TG, TRG, SFG	H375_2380	Identified by RNA-seq	This study
34	326339	326637	299	F	Cis	TG, TRG, SFG	H375_2470	Identified by RNA-seq	This study
35	334531	334333	199	R	Cis	TG, TRG, SFG	H375_2560	Identified by RNA-seq	This study
36	342505	342292	214	R	Cis	TG, TRG, SFG	H375_2630	Identified by RNA-seq	This study
37	344054	343930	125	R	Trans	Rp	>/ < / <	Identified by RNA-seq	This study
38	366166	366242	77	F	Trans	Rp	>/>/>	Identified by RNA-seq	This study
39	371859	371506	353	R	Trans	TG	>/ < / <	Predicted as SIPHT #10	Schroeder et al., 2015
40	373780	373983	204	F	Cis	TG, TRG, SFG	H375_2910	Identified by RNA-seq	This study
41	378222	378333	112	F	Cis	TG, TRG, SFG	H375_2940	Identified by RNA-seq	This study
42	407851	407640	212	R	Trans	Rp	>/ < / <	Identified by RNA-seq	This study
43	427133	426907	227	R	Trans	Rp	>/ < />	Identified by RNA-seq	This study
44	457001	456876	125	F	Trans	TG	>/>/>	Predicted as SIPHT #9	Schroeder et al., 2015
45	458370	458555	227	R	Trans	TG	>/ < />	Identified by RNA-seq	This study
46	462025	462293	268	F	Cis	TG, TRG, SFG	H375_3670	Identified by RNA-seq	This study
47	481518	481833	316	F	Cis	TG, TRG, SFG	H375_3890	Identified by RNA-seq	This study
48	494600	494387	214	R	Trans	Rp	</</<	Identified by RNA-seq	This study
49	504250	504506	256	F	Trans	Rp	>/>/>	Identified by RNA-seq	This study
50	514132	513893	240	R	Trans	TG	</</<	Identified by RNA-seq	This study
51	531332	531187	146	R	Trans	Rp	>/ < / <	Identified by RNA-seq	This study
52	535456	535256	201	R	Trans	Rp	>/ < / <	Identified by RNA-seq	This study
53	558930	558719	212	R	Trans	Rp	>/ < / <	Identified by RNA-seq	This study
54	644329	644199	130	R	Trans	Rp	</</<	Predicted as SIPHT #6	Schroeder et al., 2015
55	659164	659057	107	R	Trans	Rp	</</<	Predicted as SIPHT #5	Schroeder et al., 2015
56	669521	669772	252	F	Trans	Rp	>/>/ <	Identified by RNA-seq	This study
57	697106	696934	173	R	Trans	Rp	>/ < / <	Identified by RNA-seq	This study
58	812662	812412	251	R	Trans	Rp	</</<	Identified by RNA-seq	This study
59	830223	830416	194	F	Trans	Rp	>/>/ <	Identified by RNA-seq	This study
60	844283	844606	324	F	Trans	Rp	>/>/>	Identified by RNA-seq	This study
61	859237	859436	200	F	Trans	Rp	>/>/>	Identified by RNA-seq	This study
62	909871	910281	411	F	Cis	TG, TRG, SFG	H375_7470	Identified by RNA-seq	This study
63	925625	925929	305	F	Trans	Rp	</>/<	Identified by RNA-seq	This study
64	958095	957827	269	R	Trans	Rp	</</<	Identified by RNA-seq	This study
65	968393	968664	272	F	Trans	Rp	>/>/>	Identified by RNA-seq	This study
66	973818	973352	467	R	Trans	TG	>/ < />	Identified by RNA-seq	This study
67	998167	997927	240	R	Trans	Rp	>/ < / <	Predicted as SIPHT #25	Schroeder et al., 2015
68	1039473	1039278	195	R	Trans	Rp	</</<	Predicted as SIPHT #24	Schroeder et al., 2015
69	1046344	1046671	328	F	Trans	Rp	</>/<	Identified by RNA-seq	This study
70	1105018	1104959	59	F	Trans	Rp	>/>/>	Predicted as SIPHT #23	Schroeder et al., 2015

a *RNA sequencing data demonstrates that R. prowazekii strain Breinl encodes for at least 58 novel candidate sRNAs. Each of these candidates are listed here. The sRNA number, the approximate start location based on the start of the RNA sequencing reads, the approximate stop location based again on the RNA sequencing reads, the nucleotide size, the sRNA carrying strand, the nature of sRNA, and its homology to other Rickettsia species are shown. The column labeled as “Strand Orientation” refers to the orientation of the upstream gene, the trans-acting sRNA, and downstream gene, respectively. For cis-acting sRNAs, the corresponding ORF is listed in this column (F, forward; R, reverse; Rp, R. prowazekii only; TG, typhus group (both R. prowazekii and R. typhi); TRG, transitional group; SFG, spotted fever group; >, sense strand; <, anti-sense strand). Sequences may not be found in all species of a particular group*.

RNA sequencing of HMECs infected with *R. prowazekii* for 3 h (to enable entry and infection) resulted in ~42 to 46 million total reads, whereas 27 to 29 million total reads were obtained for RNA isolated from cells infected for 24 h. Of these, ~930,000 to 2 million reads mapped to the *R. prowazekii* genome at 3 h and a total of 1 to 4 million reads corresponded to *R. prowazekii* at 24 h. This is in agreement with a recent demonstration that intracellular organisms, such as *Rickettsia* species, constitute only 5% of extracted total RNA, whereas the remaining 95% belongs to eukaryotic host cells. Further analysis reveals that about 95% of bacterial RNA is comprised of rRNAs and tRNAs and only the remaining 5% includes mRNAs and sRNAs. This, in essence, translates to a ratio of ~1:400 bacterial mRNAs and sRNAs in the preparations of total cellular RNA from infected host cells (Westermann et al., 2012). Despite efficient removal of eukaryotic polyadenylated transcripts and ribosomal RNAs through microbial enrichment protocols, the process is limited in removing tRNAs, eukaryotic noncoding RNAs, and mitochondrial RNAs, resulting in interference. Consequently, only 2–5% of extracted total RNA generally maps to intracellular bacterial genomes (Westermann et al., 2012).

**Figure 1:**
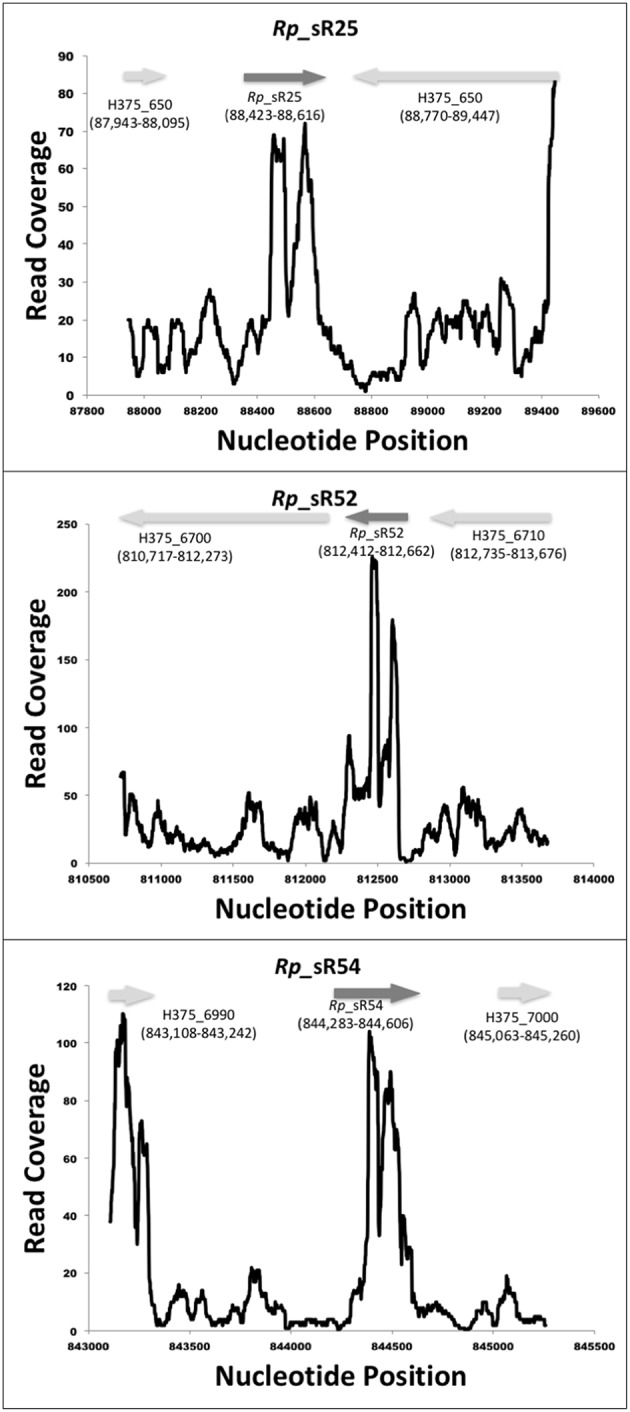
**Identified novel trans-acting candidate sRNAs**. Shown are the coverage plots for selected trans-acting sRNAs. Nucleotide positions within the genome are indicated on X-axis and the Y-axis displays the number of reads for that particular nucleotide position. The dark gray arrow represents the small RNA. The light gray arrows represent the orientation of upstream and downstream ORFs, respectively.

**Figure 2:**
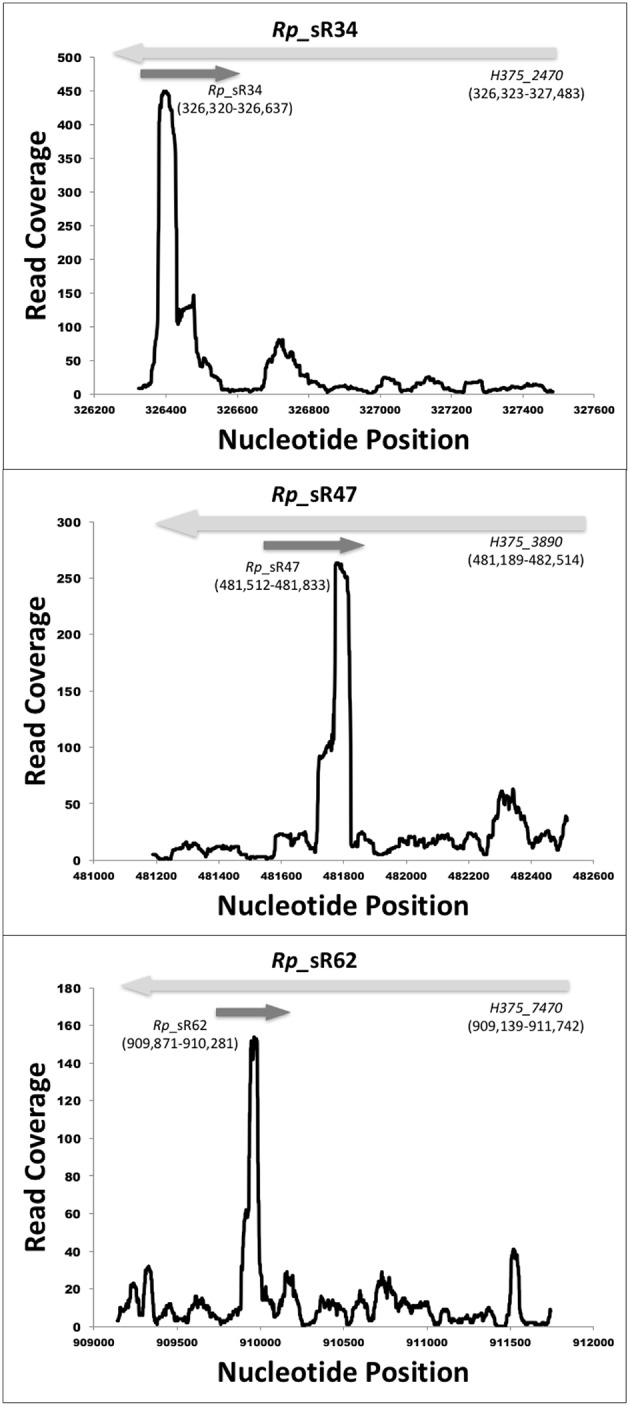
**Identified novel cis-acting candidate sRNAs**. Shown are the coverage plots for selected cis-acting sRNAs. Nucleotide positions within the genome are indicated on X-axis and the Y-axis displays the number of reads for that particular nucleotide position. The dark gray arrow represents the small RNA. The light gray arrows represent the orientation of the respective ORF.

RNA-Seq based search of the transcriptome identified a total of 70 candidate trans-acting (intergenic) and cis-acting (antisense) sRNAs that were either expressed at 3 and/or 24 h post-infection (Table 1). All sequences are available in the Bacterial Small RNA Database (Li et al., 2013) and GenBank (accession number KX215777 through KX215846). A representative selection of RNA read coverage plots for trans- and cis-acting sRNAs is presented in Figures 1, 2, respectively. Amongst the newly identified candidates, 35 candidates were trans-acting and another 23 were cis-acting sRNAs. The sizes of the candidates identified ranged from 59 to 585 bp, with an average 233 bp. Interestingly, four trans-acting candidates (named R*ickettsia* p*rowazekii* small RNAs [*Rp*_sR]5, *Rp_*sR45, *Rp*_sR50, and *Rp*_sR66) were found to have sequence homology to other rickettsial species. All four had strong (~90%) homology to the typhus group *R. typhi*, while candidate *Rp*_sR5 also shared strong homology to *R. felis*, a transitional group species. Otherwise, all remaining trans-acting sRNAs were unique to *R. prowazekii*. With the exception of two candidates (*Rp*_sR3 and *Rp*_sR18), cis-acting sRNA candidates generally shared considerable homology to rickettsial species outside of the typhus group. The corresponding ORF for *Rp_*sR3 is H375_160 (a transcription-repair coupling factor), which is present in all other sequenced *R. prowazekii* strains. For *Rp_*sR18, the corresponding ORF is a single-stranded DNA-specific exonuclease (H375_870), which is also conserved in other *R. prowazekii* and *R. typhi* strains. Considering that the intergenic regions are more likely to undergo dynamic changes in their sequences and cis-acting sequences tend to remain conserved by virtue of their location on the anti-sense strand of an ORF, the high number of candidates sharing homology outside of the typhus group is not surprising. Accordingly, 17 cis-acting sRNAs were present on the ORFs conserved in all rickettsial species, and 4 sRNAs were encoded from an antisense strand representing hypothetical and ORFan proteins (ORFs with no known homologs within current databases). Interestingly, candidate *Rp*_sR12 had no homology to the spotted fever group, but it did share homology to the ancestral group, typhus group, and transitional group. Further, candidate *Rp*_sR28 had homology to the typhus group and transitional group, but no homology was found in the ancestral group or the spotted fever group. Candidate *Rp*_sR22 shared homology to the typhus group and the spotted fever group, but not the transitional group. It is important to note that homology was not necessarily observed for all rickettsial species of a particular group. For example, homology in candidate *Rp*_sR22 was found in the spotted fever group species *R. montanesis* and *R. japonica*, but not *R. rickettsii* or *R. conorii*.

### Experimental validation of candidate sRNAs

**Figure 3:**
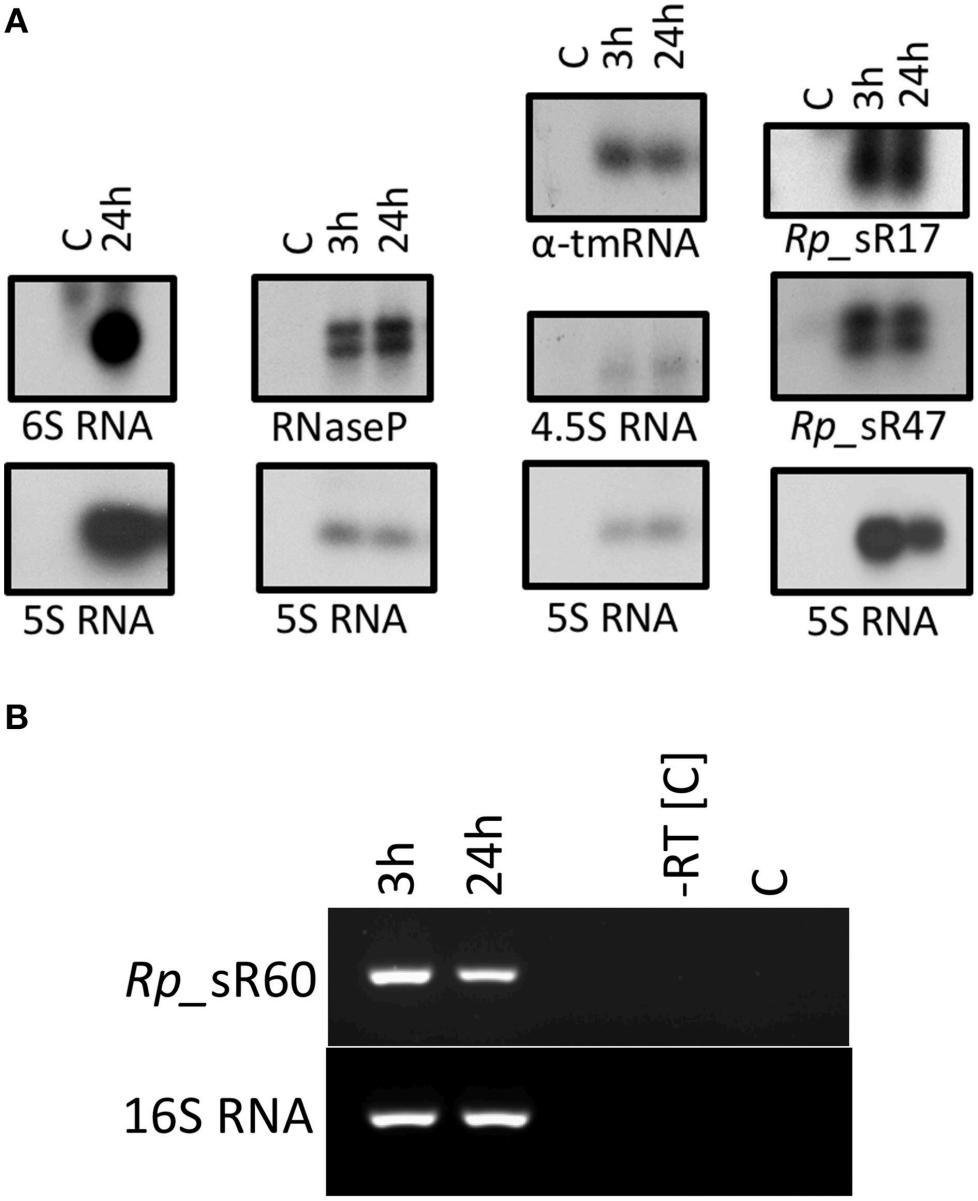
**(A)** Northern blot analysis. A representative image of the Northern blots for candidate sRNAs is shown. Northern blot analysis was performed using strand-specific sRNA probes radiolabeled with [α-^32^P] UTP. All blots included RNA samples from control (uninfected HMECs) and those infected for 3 and 24 h with *R. prowazekii*. The blot for 6S RNA only included RNA isolated from HMECS that were either left uninfected or processed at 24 h post-infection. **(B)** Representative gel image showing the expression of sRNA *Rp*_sR60 during the infection of HMECs at 3 and 24 h post-infection.

To verify expression of candidate sRNAs, Northern blots were carried out with strand-specific [α-^32^P] UTP-labeled RNA oligonucleotide probes specific to the corresponding candidate with enriched rickettsial RNA from *R. prowazekii*-infected HMECs at 3 and 24 h post-infection. Two trans-acting sRNAs (*Rp*_sR17 and *Rp*_sR60) and two cis-acting sRNAs (*Rp*_sR34 and *Rp*_sR47) identified from the RNA-Seq data were selected on the basis of their genomic location, number of RNA reads, strand orientation, as well as the orientation of corresponding upstream and downstream ORFs. Candidate *Rp*_sR67 [identified earlier with the SIPHT program as Candidate #25 (Schroeder et al., 2015)] was also selected for verification via Northern blot analysis. In addition, four other highly conserved bacterial sRNAs were also chosen. These included 6S RNA (*ssrS*), 4.5S RNA (*ffs*), RNaseP_bact_a, and α-tmRNA (*ssrA*). 6S RNA was only probed at 24 h post-infection as our previous data demonstrated that rickettsial 6S RNA was most abundantly expressed at this time-point (Schroeder et al., 2015). Strong and distinct RNA bands were observed for six probed sRNAs except for α-tmRNA, which had a light but distinct band (Figure 3A). *Rp*_sR34 and *Rp*_sR60 could not be detected by Northern blot analysis. Interestingly, the blots for RNaseP_bact_a, *Rp*_sR17, and *Rp_*sR47 revealed multiple bands, suggesting processed sRNA transcripts. As these bands are relatively similar in size, the fact that we did not see these differences in the RNA-Seq data is not surprising since the libraries were generated from both primary and processed transcripts. Because the RNA probes utilized in our experiments were strand-specific and the detected signals were at their predicted sizes, the Northern blot analysis validates and confirms independent transcription of the candidate sRNAs during host cell infection.

Since *Rp*_sR60 was not detected using Northern blot, we performed RT-PCR as an alternative approach to confirm its expression. Total RNA from *R. prowazekii-*infected HMECs clearly demonstrated expression of *Rp_*sR60 at both 3 and 24 h post-infection. Figure 3B shows a representative agarose gel with 16S RNA serving as an endogenous control. A similar approach was not amenable for the detection of *Rp_*sR34 because of its cis-acting nature and the possibility of confounding amplification of its corresponding ORF transcript. These results, nevertheless, provide clear evidence for the expression of *Rp_*sR60 during *R. prowazekii* infection of HMECs.

### Nucleotide sequencing of validated sRNAs by RLM-RACE

**Figure 4:**
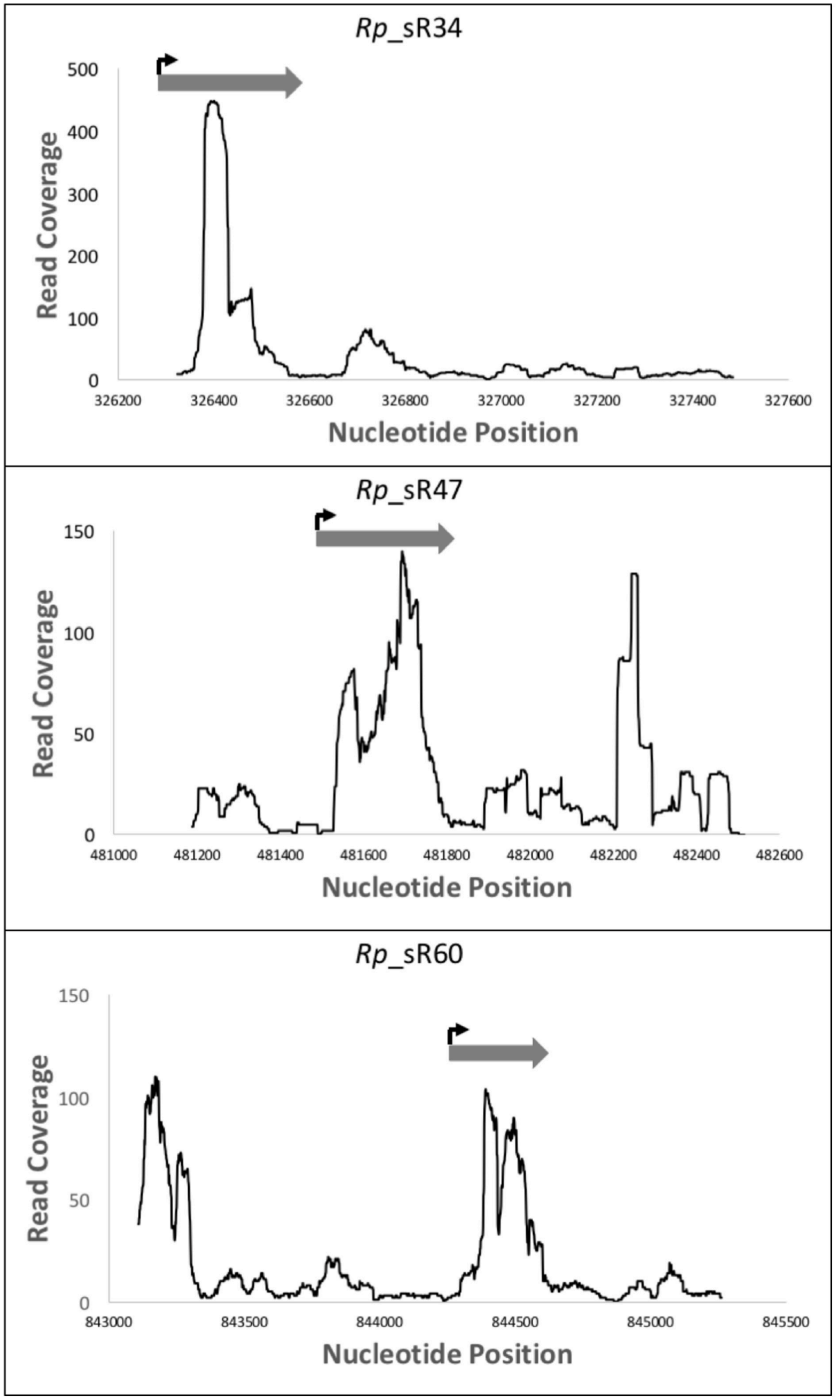
**RLM-RACE transcription start site compared to RNA-Seq read coverage**. This representation demonstrates the location of the sRNAs transcription start sites as determined by the sequencing data from 5′ RLM-RACE. These data have been overlaid with the mapping reads obtained from RNA-Seq. Nucleotide positions within the genome are indicated on X-axis and the Y-axis displays the number of reads for that particular nucleotide position. The dark gray arrow represents the small RNA as defined by the RNA-Seq reads, while the bent arrow represents the location of the transcription start site as determined by the 5′ RLM-RACE sequencing.

Northern blot analysis only provides information regarding the levels of expression and the transcript size, but is unable to reveal the exact transcription start site. Therefore, to determine the transcription start sites of confirmed novel sRNAs (*Rp*_sR17, *Rp*_sR34, *Rp*_sR47, *Rp*_sR60, and *Rp*_sR67) and 6S RNA, 5′ RLM-RACE was employed. RLM-RACE provides a distinct advantage over the traditional 5′ RACE method because it only amplifies primary transcripts, and not processed RNAs, for subsequent sequencing, which then permits the determination of an exact transcription start site. The TSSs for the novel *R. prowazekii* sRNAs, confirming the potential for their independent expression, are listed in Table 2. Furthermore, the transcription start sites deciphered from the RLM-RACE approach were found to correspond to transcriptomic data from RNA-Seq experiments and to closely align with the initiation loci of sRNAs expression (Figure 4). This correlation, thus, further supports independent expression of the *Rp*_sRs during infection of target host cells.

**Table 2:** **Identified transcription start sites determined by RLM-RACE and associated promoter motifs**.

**sRNA**	**−10 Box**	**−10 Position (bp)**	**−35 Box**	**−35 Position (bp)**	**Start site**
17	TGCTTTTAT	−93	TTGCAA	−114	105,761
34	CTTTATAAT	−45	TTGCTA	−69	326,320
47	AATTAAAAT	−65	TGGATA	−89	481,512
60	AGTTACTAT	−43	TTGGTG	−65	844,442
67	AATTATAAT	−36	TTGCAT	−56	998,424
6S	CCTTACTCT	−77	TTATAA	−94	934,943

Using the RLM-RACE sequencing data, the −10 motif and the −35 motif for the σ^70^ promoter were predicted using BPROM. The table lists the predicted −10 nucleotide sequence, the −10 start position upstream from the transcription start site, the −35 nucleotide sequence, and the −35 start position upstream from the transcription start site for the confirmed rickettsial sRNAs. The “Start Site” is the transcription start Site determined by RLM-RACE.

### Molecular analysis of 6S RNA and novel *R. prowazekii* sRNAs

**Figure 5:**
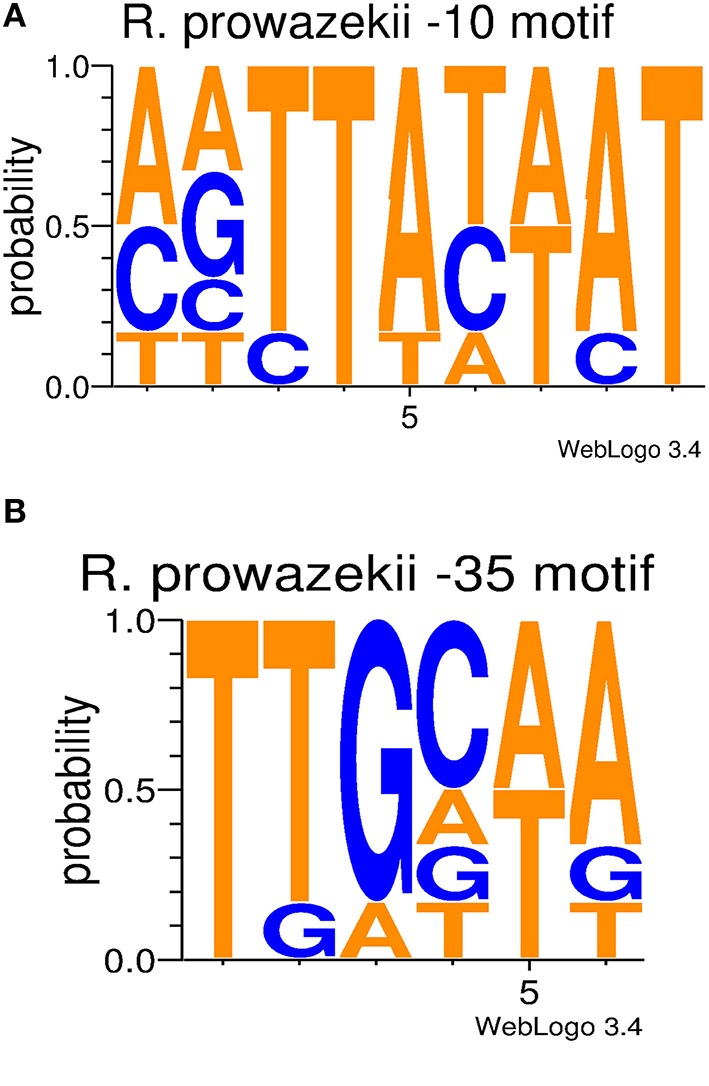
**sRNA promoter frequencies**. Conservation diagrams illustrating the probability of a nucleotide in a specific promoter motif position based on the confirmed sRNAs. Panel **(A)** shows the −10 promoter motif, while Panel **(B)** represents the −35 promoter motif. The predicted −10 motifs are similar to the *E. coli* consensus sequence (TATAAT). On the other hand, the −35 motifs differ in comparison to the *E. coli* consensus sequence (TTGACA).

In order to analyze each confirmed sRNA for potential promoter motifs (−10 and −35 sites), 150 nucleotides upstream of the identified transcription start site were submitted to the web-based program, BPROM. This range was chosen keeping in mind that ~80% of known σ^70^ promoters in *Escherichia coli* are located within 150 nucleotides of the transcription start site (Huerta and Collado-Vides, 2003). Upon predicting σ^70^ promoters for all novel sRNAs and 6S RNA, a deeper analysis demonstrated that the −10 and −35 motifs were an average of 60 nucleotides and 82 nucleotides upstream of the transcription start site, respectively. This distance ranged from 36 to 93 bp upstream for the −10 motif and 56 to 114 bp upstream for the −35 motif (Table 2). For *E. coli*, the optimum distance between the −10 and −35 motifs has been reported to be 17 ± 1 nucleotides (Harley and Reynolds, 1987; Mitchell et al., 2003), but our data indicate that the average distance for *R. prowazekii* is 21 nucleotides. This result is in close agreement with our previous findings for sRNAs predicted by SIPHT in spotted fever and typhus group rickettsiae (Schroeder et al., 2015). The predictions, which have a consensus of TATAAT, are identical to accepted −10 consensus sequence for *E. coli* (Harley and Reynolds, 1987). The first and second position of the −10 motif is conserved with T and A at 100 and 85%, respectively. The third and fourth positions are ~50% T and A. The fifth position is 85% A, while the sixth position is 100% T. On the other hand, the rickettsial −35 motif, predicted as TTGCAA, has notable differences compared to the −35 consensus sequence for *E. coli*, reported as TTGACA (Harley and Reynolds, 1987). For the rickettsial sRNAs, the first three positions are TTG nearly 82 to 100% of the time. The fourth position is a C with ~50% probability as opposed to an A in *E. coli* consensus sequence. The fifth position for *E. coli* is C, but it is A or T in *R. prowazekii*. The final position was an A with an approximate probability of 70% (Figure 5).

### Candidate sRNA target identification

Two independent programs, TargetRNA2 and IntaRNA, were used to further understand the regulatory roles of the confirmed novel trans-acting sRNAs (*Rp*_sR17, *Rp*_sR60, and *Rp*_sR67). These programs identify the sRNA:mRNA interactions by assessing base pairing potential based on a Smith-Waterman dynamic and by assessing interaction sites and seed regions, respectively (Busch et al., 2008; Tjaden, 2008). By using TargetRNA2, a total of 72 protein-coding targets were identified to be potentially regulated by sRNAs. Conversely, IntaRNA predicted a total of 122 targets with a *p* ≤ 0.05. A detailed analysis revealed common target predictions by both programs (Table 3). For *Rp*_sR60 and *Rp*_sR67, a total of 7 and 6 common protein targets were found, respectively. Three common targets predicted for *Rp*_sR67 have functional roles in tRNA synthesis and another one participates in ATP synthesis, while the other common targets were hypothetical or uncharacterized proteins. Interestingly, *Rp*_sR60 has two protein-coding mRNA targets involved in ribosomal protein synthesis and three targets involved with cell metabolism. In contrast, *Rp*_sR17 has a single common predicted target, namely protein translocase subunit, *secD*. This protein facilitates secretion across the inner membrane of Gram-negative bacteria. The target genes regulated by each sRNA are listed in Supplemental Table S1. Targets for the cis-acting sRNAs were not predicted as previous data demonstrate that cis-acting sRNAs tend to interact with their associated ORFs. This suggests the potential for *Rp*_sR34 interaction with H375_2470, a hypothetical protein, whereas *Rp*_sR47 may base-pair and regulate the expression of H375_3890, an ORF encoding for a carboxyl-terminal protease from the complementary strand. Although a large percentage of *R. prowazekii* genes either code for hypothetical proteins or remain uncharacterized, a majority of predicted mRNA targets for novel sRNAs have an assigned function. It is reasonable to posit, therefore, that sRNAs may have regulatory influence on at least some of the predicted targets and are likely to be functional in *R. prowazekii*.

**Table 3:** **Prediction of target genes using TargetRNA2 and IntaRNA**.

**sRNA candidate name**	**Total number of target genes predicted by TargetRNA2**	**Total number of target genes predicted by CopraRNA**	**Number of common targets predicted by both programs**	**List of target genes predicted by both programs**	**Protein name**
*Rp_*sR17	19	44	1	H375_290	Protein translocase subunit SecD
*Rp_*sR60	23	40	6	H375_7590	3-oxoacyl-[acyl-carrier-protein] synthase 2
				H375_4980	SSU ribosomal protein S7p (S5e
				H375_3020	Putative TolA protein
				H375_1850	Phosphatidate cytidylyltransferase
				H375_8280	Putative cytochrome c-type biogenesis protein related to CcmF
				H375_8880	LSU ribosomal protein L6p (L9e)
*Rp_*sR67	30	37	7	H375_8490	Methionine–tRNA ligase
				H375_6610	Alanine–tRNA ligase
				H375_4810	Hypothetical protein H375
				H375_2550	Reductase
				H375_1110	tRNA pseudouridine synthase B H375
				H375_3730	Uncharacterized protein RP244 of Rickettsia
				H375_6090	ATP synthase subunit c H375

Listed are the common set of targets predicted by both TargetRNA2 and IntaRNA. The total number of targets predicted by TargetRNA2 is shown next to the total number of targets predicted by IntaRNA. The number of common targets is documented next to a list of those common targets and their functions.

## Discussion

Despite recent developments and tremendous progress in the identification of bacterial sRNAs and discoveries uncovering their novel roles as important post-transcriptional regulators, rickettsial sRNAs remain largely unknown and uncharacterized. A recent study from our laboratory reported on the predictive analysis of rickettsial sRNAs by SIPHT and an initial confirmatory analysis of their presence and expression in infected host cells by RT-PCR (Schroeder et al., 2015). Here, we report on a transcriptome wide analysis focused on the identification of entire repertoire of both trans-acting and cis-acting sRNAs in the virulent Breinl strain of *R. prowazekii* expressed during the infection of host cells *in vitro*. We have identified a sum of 58 novel sRNAs through RNA-Seq and confirmed the expression of four sRNAs along with 6S RNA, α-tmRNA, RNaseP_bact_a, and 4.5S RNA. We have further performed RLM-RACE to identify their transcription start sites and computational promoter analysis on the confirmed sRNAs. Also, target predictions using two independent programs, TargetRNA2 and IntaRNA, have revealed probable mRNA targets for each trans-acting sRNA.

Most sRNAs described to date have been found in model organisms, such as *Escherichia coli, Salmonella enterica* serovar Typhimurium, and *Staphylococcus aureus* (Sharma and Heidrich, 2012). These sRNAs control gene expression as determinants of bacterial virulence, stress response, motility, biofilm formation, host immune responses, and other necessary functions and thus play a critical role in bestowing the adaptation skills that allow the pathogen to launch a quick response to environmental changes (Chabelskaya et al., 2010; DiChiara et al., 2010; Sharma and Heidrich, 2012; Thomason et al., 2012). Although, not yet characterized in as much detail, small RNAs are now beginning to be described in a wide range of α-proteobacteria, including *Coxiella, Bartonella*, and *Rickettsia* species (Warrier et al., 2014; Schroeder et al., 2015; Tu, 2015).

Previous studies demonstrate that *R. prowazekii* relies on leaky transcriptional termination for host adaptation and survival (Woodard and Wood, 2011). Therefore, to ascertain whether or not our candidates represent independent transcripts or are simply the result of leaky termination, we calculated the MEV for each of the sRNA candidates that were in the same orientation as the closest upstream ORF and compared them to the MEVs calculated for the respective 50-base flanking regions. Of the 58 candidate sRNAs, 22 candidates were identified to be in the same orientation as the closest upstream ORF. Each candidate had an MEV of ≥5 when compared to the surrounding 50-base flanking regions with the only exception of candidate *Rp*_sR17, which had an MEV of ≥2 when compared to its flanking region. Even though it did not surpass the cut-off threshold of five-fold used in this study, expression of sRNA candidate *Rp*_sR17 is quite likely to be independent from its neighboring ORFs and the low MEV may be due to its lower level of expression during infection of HMECs. The remaining 33 candidate sRNAs were in an orientation opposite their closest upstream ORF. For this situation, it is reasonable to expect that evidence for sRNA expression is not an outcome of leaky termination, as the reads corresponding to candidate sRNAs map onto the opposite strand. These data, thus, support the notion that identified candidate sRNAs are not the consequence of leaky termination, but instead represent bona fide transcripts expressed independently of their flanking ORFs.

In this study, we have identified a total of 32 novel sRNAs to be specifically present in *R. prowazekii* genome, while only 26 sRNAs were shared among species belonging to other rickettsial groups (Table 1). The occurrence of vast number of *R. prowazekii-*specific sRNAs is not surprising, considering that rickettsial genomes have undergone genome degradation and rearrangements resulting from horizontal gene transfer (HGT), transposon mutagenesis, and pseudogenization (Andersson et al., 1998; Fournier et al., 2009; Gillespie et al., 2012). For instance, the genome of *Rickettsia* endosymbiont of *Ixodes scapularis* (REIS) is overrun by mobile genetic elements and conjugative plasmids resulting in acquisition of nearly 32% of the ORFan genes unique to REIS. It is presumed that large tracks of unique genes along with their IGRs may have been acquired from other unknown organisms with AT rich genomes similar to rickettsiae (Gillespie et al., 2012). Species-specific sRNAs arising from genome rearrangements, deletions, and point mutations have been reported in several bacteria, such as *E. coli* and *S. entrica* serovar Typhimurium (Mendoza-Vargas et al., 2009; Raghavan et al., 2015).

Although next generation sequencing yields convincing data suggesting expression of selected *R. prowazekii* sRNAs during infection of HMECs, an important next step is to validate their expression via independent experimental methods considered to be the “gold standard” in the fields of RNA biology in general and bacterial sRNAs in particular. Accordingly, we chose four novel sRNA candidates for validation through Northern blot analysis with strand-specific RNA probes and 5′ RLM-RACE. Previously, qRT-PCR based analysis of *R. prowazekii-*infected HMECs had demonstrated that 6S RNA expression was significantly higher at 24 h post-infection in comparison to the same at 1.5h (Schroeder et al., 2015). In the present study, Northern blotting confirmed abundant expression of 6S RNA at 24 h post-infection as expected. Importantly, Northern blots and RT-PCR further revealed clear expression of three selected novel sRNA candidates (*Rp_*sR17, *Rp_*sR47, and *Rp_*sR60) at 3 and/or 24 h post-infection. In regards to the well-known sRNAs (RNaseP_bact_a, α-tmRNA, 4.5S RNA, and 6S RNA), there was clear expression at 3 and 24 h post-infection. The successful sequencing and determination of transcriptional start sites for each novel sRNA further demonstrates that *R. prowazekii* does, indeed, express these sRNAs during host cell infection. When the RLM-RACE data is overlaid with the RNA-Seq data, the transcription start sites, as determined by RLM-RACE, correspond to the start of the sequencing reads with the exception of *Rp*_sR60, in which case the transcription start site determined by RLM-RACE mapped toward the middle of the observed RNA-Seq reads. This could potentially be an outcome of: (i). occurrence of two novel small RNAs that may either have a significant overlap or may be located within extreme close proximity of each other in the *R. prowazekii* genome, (ii). presence of an unannotated ORF upstream of the sRNA TSS, and (iii). *Rp*_sR60 could be a riboswitch containing sRNA. Translation of the sequence upstream of the TSS identified by RACE revealed the existence of a predicted hypothetical ORF between positions 844285 and 844416. However, the –10 and –35 motifs were absent upstream of this predicted ORF. Riboswitch sRNAs are present in the 5′ untranslated region of the coding genes and regulate the expression of the downstream gene in response to metabolic stimuli. Several riboswitches have been well characterized in different bacterial species. *Bartonella*, a facultative intracellular pathogen, is shown to express at least 9 riboswitch sRNAs (Brt 1-9) upstream of different helix-turn-helix XRE genes (Tu, 2015). Most recently, EutX and Rli55 sRNAs belonging to *Enterococcus faecalis* and *Listeria monocytogenes*, respectively, are shown to contain riboswitches regulated by ethanolamine. The EutX is expressed as two independent transcripts depending on the presence or absence of ethanolamine and cofactor adenosylcobalamin (AdoCbl) (DebRoy et al., 2014; Mellin et al., 2014). Further studies on the *Rp*_sR60 genomic region are, therefore, necessary to resolve the differences in our RNAseq and RLM-RACE based annotation of TSS. Taken together, the results from Northern blot analysis and RLM-RACE sequencing as two independent approaches not only substantiate the presence and expression of the sRNA candidates under investigation in *R. prowazekii*, but also allow for the determination of their transcriptional start sites within the genome.

**Figure 6:**
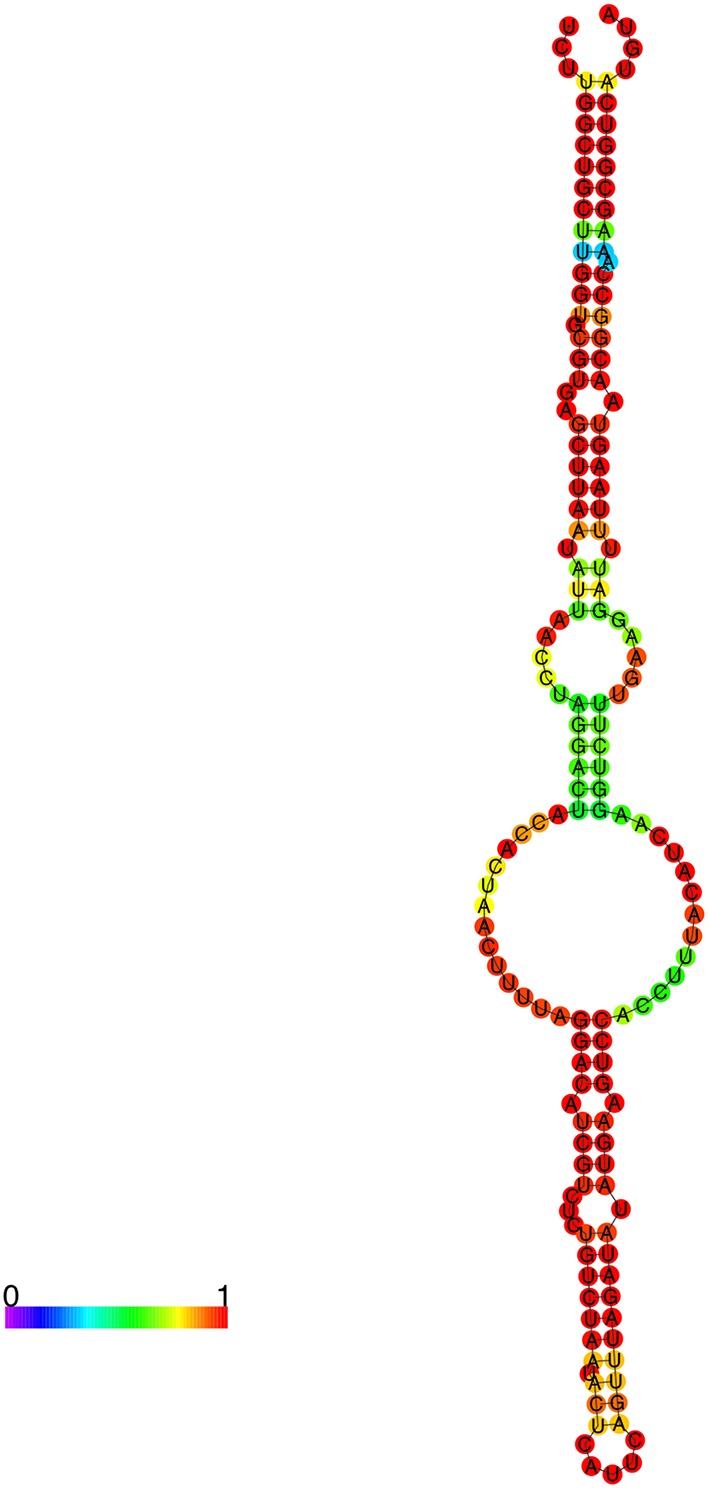
**Predicted secondary structure of *R. prowazekii* 6S RNA**. Predictive analysis of the secondary structures of identified sRNAs was accomplished using RNA-fold. The color palette represents base-pairing probability from 0 to 1 (purple to red). This secondary structure of *R. prowazekii* 6S RNA resembles the previously reported consensus secondary structure of bacterial 6S RNA.

Using the web-based program RNAfold (Hofacker, 2003), a prediction of the secondary structure for *R. prowazekii* 6S RNA based on the RLM-RACE data and the RNA-Seq data reveals that it is quite similar to other published bacterial 6S RNAs (Figure 6) (Barrick et al., 2005). The structure is composed of a single central strand, the ends of which contain either a closed stem or a terminal loop. The purpose of the central bubble is to mimic an open promoter complex on a DNA template (Wassarman and Storz, 2000; Wassarman, 2007). This bubble is responsible for RNA polymerase binding to the 6S RNA molecule and eventually becoming sequestered. Based on its pattern of expression and the predicted secondary structure, it is quite likely that 6S RNA in *R. prowazekii* is functional. We have also predicted the secondary structures of novel rickettsial sRNAs using the determined start site and a predicted termination location (Supplemental Figure S1). As an example, *Rp*_sR67 predictably contains three arms organized around a central bulge. The sRNA secondary structures are predominantly projected as the indicators of evolutionary conservation/relationships and structure-function prediction based on the thermodynamics of stable structures (Mathews et al., 2010). Distinct mechanisms of regulation are employed by *Vibrio harveyi* quorum sensing Qrr sRNA depending on the base pairing of the nucleotides in different stem loops that are involved in interactions with the target gene. The binding of luxM and aphA target genes to the first stem loop of Qrr sRNA results in the degradation of Qrr sRNA. The strong binding of luxO to the second stem loop, on the other hand, leads to the sequestration of the sRNA and catalytic repression of Qrr occurs following relatively weak interactions between the sRNA and luxR mRNA (Feng et al., 2015). The prediction of multiple target genes regulated by *R. prowazekii* sRNA repertoire (Table 3) coupled with the existence of complex RNA fold structures such as several stem loops suggests the potential for their involvement in the diverse regulatory mechanisms. Hence, the details of their functions and the mechanisms of action are critically important topics indeed for further detailed investigations.

The discovery of small RNAs in a wide range of bacterial pathogens and the obvious need for further definition of their functions served as a stimulus for the development of algorithms to predict their possible targets. As such, a variety of computational programs have been developed for the prediction of small RNA targets. For this study, we chose TargetRNA2 and IntaRNA to predict the potential mRNA targets for trans-acting sRNAs in *R. prowazekii*. An important consideration for trans-acting sRNAs originating from intergenic regions is that their targets can be located elsewhere in the genome and are not necessarily restricted to adjoining or neighboring ORFs. As expected, the predicted targets for *Rp*_sR17, *Rp*_sR60, and *Rp*_sR67 primarily include bacterial metabolism and other housekeeping functions. However, a confounding factor is that a number of potential targets are either hypothetical or uncharacterized genes, rendering the possible downstream functional implications of these sRNAs difficult to analyze and interpret. It must also be noted that trans-acting small RNAs require the use of a chaperone molecule to facilitate sRNA:target binding due to a limited nucleotide similarity. The most prominent among the sRNA chaperones is Hfq, a small hexameric RNA-binding protein, whose role as a post-transcriptional regulator has been recognized relatively recently (Chao and Vogel, 2010). Hfq-dependent sRNAs usually repress translation and/or increase target destruction through ribonuclease E (RNase E) activity (Chao and Vogel, 2010), although there are some examples in which Hfq-dependent sRNAs stabilize their target mRNAs. Interestingly, both typhus group (*R. prowazekii* and *R. typhi*) and spotted fever group (e.g., *R. rickettsii, R. conorii*) are not known to encode for *hfq*, nor has another chaperone molecule been described in *Rickettsia* species (Sun et al., 2002; Chao and Vogel, 2010). Nearly all members of Enterobacteriaceae encode for the *hfq* gene, but nearly 50% of other bacteria do not (Sharma and Heidrich, 2012). These include *Streptococcus, Mycobacteria, Helicobacter*, and *Chlamydia*, which all have validated small, noncoding RNAs. This suggests the use of an alternative chaperone molecule to facilitate rickettsial trans-acting sRNA interactions with target mRNAs. Indeed, potential non-Hfq chaperone molecules have been described in *Helicobacter pylori* and *Mycobacterium tuberculosis* (Basu et al., 2009; Rieder et al., 2012).

Because cis-acting sRNAs exhibit perfect nucleotide similarity, the expected target would be the corresponding ORF on the opposite strand. In this context, H375_2470 as the corresponding ORF for *Rp*_sR34 has been annotated as a hypothetical protein, and thus it is difficult indeed to hypothesize the significance or role of this sRNA for *R. prowazekii* until the function of this protein in rickettsial lifecycle has been ascertained. A BLAST search reveals that H375_2470 is conserved in rickettsial genomes; therefore, it is likely a functional protein. On the other hand, *Rp*_sR47 is antisense to a known carboxyl-terminal protease (H375_3890). Carboxyl-terminal proteases (CTPs) are a new group of serine proteases, including tail-specific proteases (TSPs), that have been recognized as critical players in bacterial protein processing (Hoge et al., 2011; Seo and Darwin, 2013). The functions of CTPs include post-translational modification, maturation, and/or disassembly/degradation of proteins performing basal physiological functions and virulence factors (Lad et al., 2007; Hoge et al., 2011). *Chlamydia trachomatis*, an obligate intracellular human pathogen, has recently been described to secrete a tail-specific protease CT441, which degrades p65 and disrupts the NF-kB pathway of host antimicrobial and inflammatory responses (Lad et al., 2007). Similarly, *R. rickettsii* interacts with inactive NF-kB of endothelial cell cytoplasm in a “cell-free” system resulting in its activation as evidenced by increased DNA-protein binding in a mobility shift assay (Sahni et al., 1998, 2003). Furthermore, *Pseudomonas aeruginosa*, an opportunistic human pathogen, relies on CtpA for normal function of type 3 secretion system (T3SS). The T3SS is essential for cytotoxicity toward host cells and is a vital virulence factor in mouse models of acute pneumonia. Conversely, up-regulation of CtpA in *P. aeuroginosa* induces the expression of an extracytoplasmic function sigma factor regulon, resulting in an attenuated phenotype in rat models of chronic lung infection (Seo and Darwin, 2013). In the facultative intracellular pathogen *Brucella suis, ctpA-*deficient strains display altered morphology, increased cell size, and partial dissociation of cell membrane from the envelope and are cleared 9 weeks post-inoculation in a BALB/c mouse model, suggesting that CtpA is critical for survival in macrophages (Bandara et al., 2005). Since our RNA-Seq data indicates simultaneous expression of both *Rp*_sR47 and H375_3890, we hypothesize that *Rp*_sR47 interaction with H375_3890 may lead to the stabilization of the transcript for efficient translation. Further evaluation of this interaction and its functional implications in *R. prowazekii* are currently ongoing.

The field of bacterial pathogenesis is rapidly evolving and expanding with enhanced appreciation of the vastness of virulence factors and effector sRNAs. Here, we report on the identification of a number of sRNAs encoded and expressed by *R. prowazekii* during *in vitro* infection of vascular endothelial cells, the primary target cell-type during human disease. We have further substantiated the potential implications of this new regulatory paradigm in rickettsial biology via determination of transcription start sites of select novel rickettsial sRNAs and predictive analysis of their target genes involved in the pathways of bacterial maintenance, growth, and survival, and mechanisms of pathogenesis. In summary, this study provides the very first glimpse of non-coding transcriptional landscape of *R. prowazekii* in target host cells.

## Author contributions

CS performed all bioinformatics analyses, *in vitro* experiments and data analysis, and prepared the draft of the manuscript; HN conceptualized the idea of sRNAs in *Rickettsia* species and assisted with the performance and analysis of all aspects; MR, KK, and YF provided assistance with the details of bioinformatics and calculation of MEVs; AS and JP assisted with the preparation of rickettsial stocks and cultures of endothelial cells; RS assisted with *in vitro* experiments and data analysis; and SS conceived of the study and participated in its design and coordination and helped to finalize the manuscript. All authors read and approved the final manuscript.

## Funding

This work was supported, in part, by a pilot project grant from the Institute for Human Infections and Immunity, the James McLaughlin Fellowship Program, and institutional support funds from the University of Texas Medical Branch, and an exploratory research grant AI115231-01A1 from the National Institute of Allergy and Infectious Diseases at the National Institutes of Health, Bethesda, MD, USA.

### Conflict of interest statement

The authors declare that the research was conducted in the absence of any commercial or financial relationships that could be construed as a potential conflict of interest.
