# Supplementary material for: Identification and Characterization of Novel Small RNAs in Rickettsia prowazekii
Source: Front Microbiol. 2016 Jun 8;7:859. doi: 10.3389/fmicb.2016.00859 (PMC4896933; doi:10.3389/fmicb.2016.00859)

Supplemental Figure S1: **Predicted secondary structure of novel sRNAs.** Predicted secondary structures for *Rp*_sR17, *Rp*_sR34, *Rp*_sR60, and *Rp*_sR67 as determined by RNA-fold. Color represents base-pairing probability from 0 to 1 (purple to red).

*Rp*_sR17

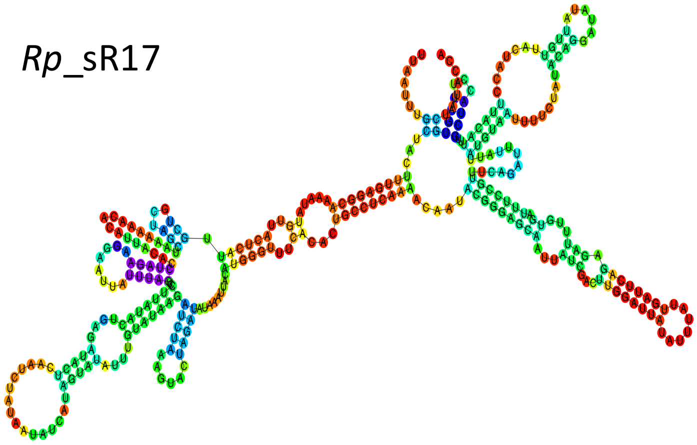

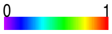

*Rp*_sR34

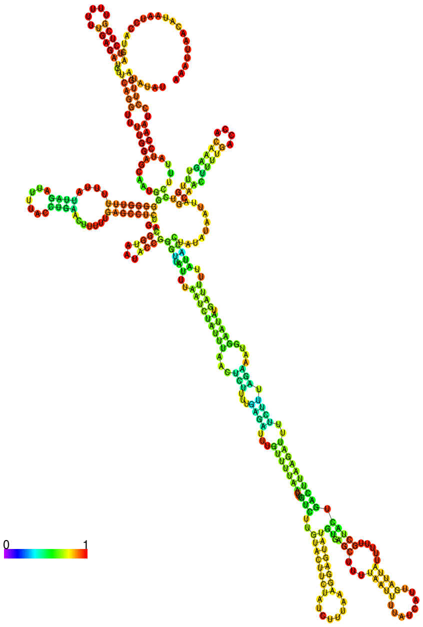

*Rp*_sR47

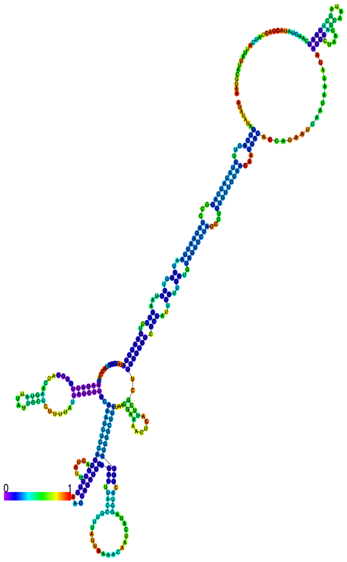

*Rp*_sR60

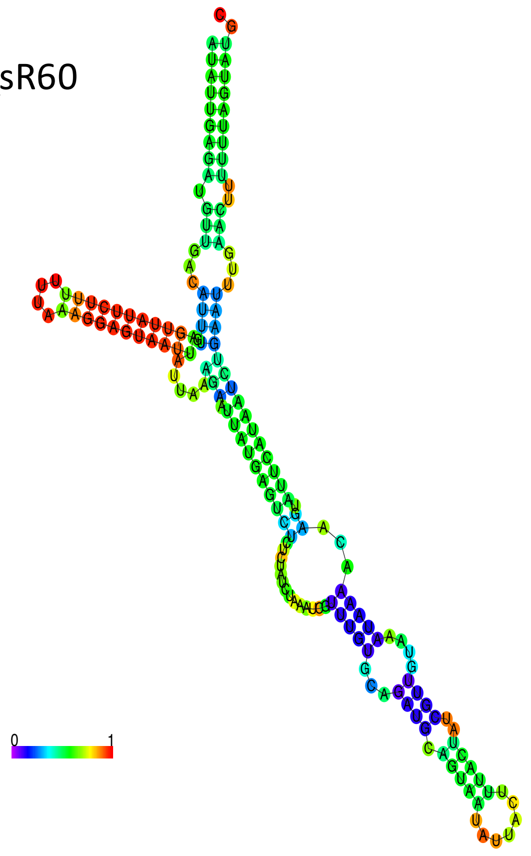

*Rp*_sR67

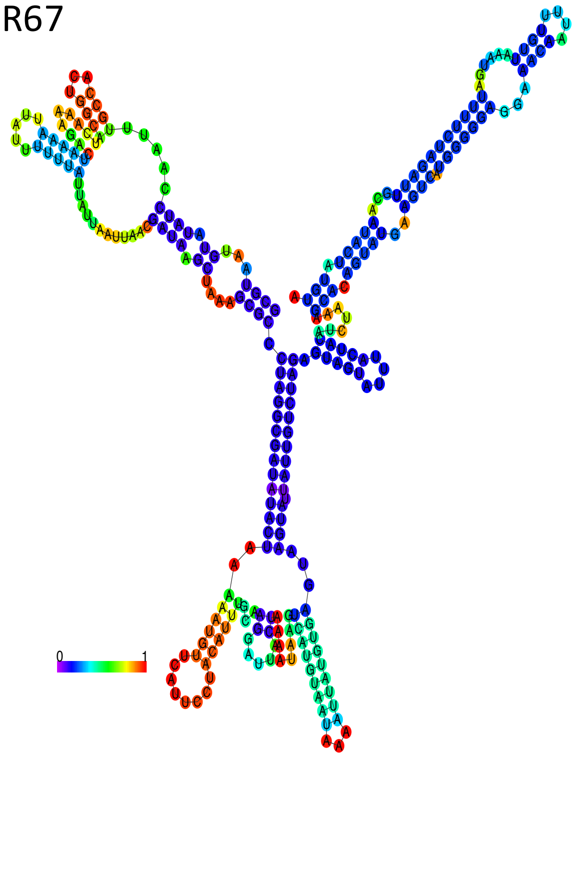

Supplement: Supplementary file 4 [file Image1.PDF]
